# Influence of climate change on adolescents and young adults’ mental health in Southern Africa: Scoping review

**DOI:** 10.4102/phcfm.v18i1.5154

**Published:** 2026-06-29

**Authors:** Mutshidzi Mulondo, Tandrea Carter, Martie Thompson, Sithembiso Ndlovu

**Affiliations:** 1 Department of Public Health, Faculty of Health Sciences, University of the Free State, Bloemfontein, South Africa; 2 Department of Public Health, Faculty of Health, Appalachian State University, Boone, United States

**Keywords:** mental health, climate change, adolescents, young adults, Southern Africa

## Abstract

**Background:**

Prioritising the mental health of adolescents and young adults during climate change is essential to their overall wellbeing. In 2021, the World Health Organization (WHO) included preventing and treating non-communicable diseases and mental health conditions as part of the 10 global health issues to track. Evidence regarding the effect of climate change on mental health conditions in Southern Africa is lacking.

**Aim:**

This scoping review aims to document the influence of climate change on adolescents and young adults’ mental health.

**Setting:**

The review was conducted according to the Preferred Reporting Items for Systematic reviews and Meta-Analyses extension for Scoping Reviews (PRISMA-ScR).

**Methods:**

There were 14 electronic bibliographic databases that were searched together with a grey literature search. Two reviewers independently screened and appraised identified articles. All relevant data were extracted and mapped according to three categories: (1) type of climate change, (2) impact and loss experience and (3) health promotion interventions.

**Results:**

We identified seven studies showing the influence of climate change on adolescents and young adults’ mental health in Southern Africa. Three (*n* = 3), 50%, of the studies appear to indicate the influence of climate change on mental health as being more long-lasting signified by symptoms of traumatisation.

**Conclusion:**

Higher rates of depression were associated with the type of climate disaster such as those found in those who experienced drought and floods.

**Contribution:**

Our review also highlighted a gap for future studies indicating health promotion interventions as being mostly social support and minimal indication of health system interventions. Future research may be necessary to provide multi-sectorial interventions of support.

## Introduction

Globally, climate change remains one of the most prevalent public health challenges and poses threats to humanity.^1,2^ It involves not only an increased frequency and severity of extreme weather events (EWEs), such as wildfires, floods, droughts, heat waves, among others, which consequently result in an increase in prevalence of diseases, shortage of food, lack or absence of clean water and overall economic hardships.^3,4,5^ Climate change is also a psychological and not just an environmental challenge.^6^ In 2021, the World Health Organization (WHO) included preventing and treating non-communicable diseases and mental health conditions as part of the 10 global health issues to track in which, among other issues, they aimed to increase community-based awareness and mental health care.^7^ Climate change and mental health have less of an obvious connection.^6^ However, there is growing literature with regard to the influence of climate change on mental health.^8,9^ People and communities are becoming more vulnerable to various mental and physical illnesses, housing issues, infrastructure issues, agricultural problems and natural ecological calamities as a result of climate change.^3^ The EWEs often result in complex variances that are associated with mental health.^4^ There are (in)direct consequences of climate-related events.^10^ Furthermore, EWEs are becoming more frequent and severe because of climate change and long-standing research has shown that natural disasters can influence mental health.^6^

Children are likely to be the population that is most affected by climate change.^6^ The EWEs are said to be the entry point in which children’s mental health and overall health is affected by climate change, particularly those who reside in at-risk areas.^11^ According to Burke et al.,^3^ young people and children are also susceptible to the consequences of climate change; however, there remains a lack of research on the impact as the body of knowledge has predominantly focused on the adult population. The WHO defines adolescence as the age between 10 years and 19 years. While there is no universally agreed upon definition for youth, the United Nations defines it as any persons between the ages 15 years and 24 years. Burke et al.^3^ further proclaim paucity of literature examining how children and the youth cope with climate change as one of the main stressors. It thus remains pivotal to conduct more research that will examine how climate change influences young people’s mental health, particularly in Southern Africa, which is susceptible to climate change as it is already warmer and drier due to its geographical location which has a subtropical climate. Furthermore, in Southern Africa, warming is increasing at a rate that is twice the global average rate, which makes the region particularly vulnerable to climate change.^12^ A special report by the Intergovernmental Panel on Climate Change (IPCC) called the Special Report on Global Warming of 1.5°C (SR1.5) identified Southern Africa as a climate change ‘hotspot’ as climate change impacts are abnormally high in relation to the global context. South Africa, which is in the southern-most point of Africa also lies on the drought-bed, which is signified by its high temperatures and low rainfall.^13^

The purpose of this scoping review was to comprehensively map the existing literature on the influence of climate change on the mental health of adolescents and young adults, synthesise the current state of knowledge and identify research gaps. There are no similar prior reviews on this topic to our knowledge. Thus, knowing the breadth and magnitude of the impacts of climate change is the first step towards designing and implementing mental health therapies to treat or prevent the mental health consequences of climate change.^10,13^

## Research methods and design

### Study design

This study employed the methodological framework by Arksey and O’Malley^14^ to conduct the scoping review. The steps to be followed were: (1) identify the research question and clear objective(s) to be addressed in the review; (2) identify relevant literature by conducting a literature search on Elton B. Stephens Company host (EBSCOhost) and Google Scholar electronic databases; (3) screen and select studies to be used in the synthesis and data extraction; (4) chart the data; and (5) collate, summarise and report the findings of the synthesis and review.^14^ Results are presented according to the Preferred Reporting Items for Systematic reviews and Meta-Analyses extension for Scoping Reviews (PRISMA-ScR) Checklist^15^ and the PRISMA 2020 guidelines.^16^ This study uses a similar methodological strategy as described in Mulondo et al.,^17^ which was conducted by the same research group.

### Inclusion and exclusion criteria

We followed the PCC (Population, Concept, Context) framework of the Joanna Briggs^18,19,20,21,22,23,24^ Institute (JBI) to convey the inclusion and exclusion criteria.^25^ The inclusion criteria included all empirical studies about mental health and climate change published about adolescents and young adults in Southern Africa between 2013 and 2023 as this provided a 10-year time period for review. All research designs were included. Relevant non-empirical works including news articles, government reports and other non-scientific publications identified within the literature search were reviewed for content and used to frame understanding of the impact of research findings. The exclusion criteria on language preference of the search were set and limited to English. Studies that did not include clear methodology (insufficient information to determine study design, study population or measures) and results were excluded. Studies that included Southern African participants whose data could not be isolated from participants from other regions of the world were also excluded.

### Search strategy

This scoping review includes literature from January 2013 to June 2023. Relevant articles and literature were searched from EBSCOhost, Academic Search Ultimate, Africa-Wide Information, APA PsycArticles, APA PsycInfo, Applied Science & Technology Source Ultimate, Cumulative Index to Nursing and Allied Health Literature (CINAHL) with Full Text, Communication & Mass Media Complete, Health Source: Nursing or Academic Edition, Humanities Source Ultimate, MEDLINE, Sociology Source Ultimate, Business Source Ultimate, GreenFILE and Google Scholar. The researchers consulted literature to ensure they include the relevant words that speak on climate change. An academic search of search words was conducted using full and partial keywords related climate change (climate* change, global warming, climate crisis, climate emergency, greenhouse gas, global heat, temperature change, severe weather, catastrophic weather and extreme weather), AND youth (adolescent, teen, young adult and youth) AND mental health (mental health, mental wellness, depression and anxiety). The university librarian assisted in finalising the key term search strategy and in obtaining relevant study documents which were not easily accessible to the researchers.

### Study selection

The screening of the studies was done by the researchers of this study. Two researchers (Tandrea Carter and Martie Thompson) screened the title and abstract, then they screened the full text of studies that could be potentially eligible. Disagreements were resolved upon discussion with a third researcher (Mutshidzi Mulondo). Seven studies met all inclusion criteria.^18,19,20,21,22,23,24^

### Data extraction and analysis

The data extraction table includes authors, year of publication, purpose of study, year study was conducted, study design, geographical location of study, age of population, measure of climate change, mental health impacts and protective factors. Data extraction was conducted by two researchers (Martie Thompson and Tandrea Carter). A research assistant conducted an initial review of the search results identifying duplicates and studies which did not meet inclusion criteria based upon article content and location of study sample. Martie Thompson and Tandrea Carter reviewed any studies identified for possible deletion and made the final determination regarding inclusion. Martie Thompson and Tandrea Carter independently reviewed the remaining articles to determine which ones met the full inclusion criteria (empirical research, location and age of study participants, assessment of the relationship of climate change and mental health). Martie Thompson and Tandrea Carter independently extracted data from the included studies and then jointly reviewed their findings to create the final data extraction table. The completed data extraction sheet has been included as a Table 1.

**TABLE 1:** Data extraction table.

Authors/Year	Study design and purpose	Age/population	Country	Source of funding	Climate change conceptualisation/measured	Mental health variables measured	Results
Sifelani I et al. (2022)	Qualitative Purpose: To understand the psychologic al impact climate-related of a disaster and the coping strategies adopted with the cyclone-induced losses.	*n* = 15 Age range: 12–16	Zimbabwe	No financial support	Climate change-related event – Cyclone Idai Event-related losses measured across four domains: (1) physical, psychological and emotional health loss; (2) basic needs loss; (3) educational loss; and (4) human life loss.	Psychological, social and physical wellbeing and coping strategies	Disaster-related losses were associated with increased distress, sadness and worry and with the onset of phobias regarding rainfall Coping Strategies: Participants used positive self-talk, positive thinking and social support to deal with disaster-related losses.
Bidassey-Minali S, et al. (2016)	Correlational Purpose: To understand the impact of increasing classroom temperatures on students’ wellbeing.	*n* = 252 Age range: 14–18	South Africa	National Research Foundation (NRF) Thuthuka fund, a Parliamentary Grant from the Council for Scientific and Industrial Research (CSIR), the South African Medical Research Council (SAMRC), the City of Johannesburg (CoJ) and Tshwane University of Technology (TUT)	Hourly indoor classroom temperature and humidity	Heat-related health impacts and perceived health symptoms including cognitive symptoms that could be related to mental health concerns – ‘low concentration’, ‘slow’ and ‘tired’.	Increased heat and humidity were negatively associated with perceived cognitive functioning
Simon T, et al. (2016)	Descriptive Purpose: To assess post-traumatic stress disorder (PTSD) on school children as a result of the floods.	*n* = 480 Age range: 8–18	Namibia	No financial support	Flooding	Post-traumatic stress disorder	The majority of children experienced PTSD symptoms after the flood. Older students had more PTSD symptoms
Prencipe et al. (2021)	Correlational Purpose: To assess associations of depression with five social determinants of health domains.	*n* = 2458 Age range: 14–19	Tanzania	Funding for the Cash Plus pilot and evaluation has been provided by Oak Foundation (#OCAY-16-73) and UNICEF. Additional funding for the evaluation was provided by the UK’s Department of International Development (DFID 203529102) and the Swedish Development Cooperation Agency (Sida G41102), both through a grant to UNICEF Office of Research – Innocenti supporting the Transfer Project. Additional funding for implementation activities was provided by Irish Aid. Additional funding for analysis and write-up of this manuscript was provided to TAJH and LP by the D.P. Hoijer Fonds, Erasmus Trustfonds, Erasmus University Rotterdam, the Netherlands and through a grant awarded by the Norwegian Research Council (project number 288638) to the Centre for Global Health Inequalities Research (CHAIN) at the Norwegian University for Science and Technology (NTNU). The funders had no role in analysis or interpretation of data.	Droughts and floods	Depressive symptoms	Experiencing droughts and floods was associated with depressive symptoms; coping or protective factors: Social support was negatively associated with depressive symptoms
Prencipe et al. (2023)	Correlational Purpose: To compare depression prevalence by extent of climate change distress and climate-sensitive living conditions.	*n* = 2053 Age range: 18–23	Tanzania	Erasmus Trustfonds, Centre for Global Health Inequalities Research, UK’s Foreign, Common wealth, and Development Office, Oak Foundation, UNICEF, UK’s Department of International Development, the Swedish Development Cooperation Agency, Irish Aid	Awareness of climate change and climate change distress	Depressive symptoms	Climate change awareness was strongly associated with climate change distress. Climate change distress was associated with greater depressive symptoms Coping or protective factors: Religious attendance was assessed and was found to be *positively associated* with climate change distress
Zeligman et al. (2020)	Correlational Purpose: To investigate if trauma symptoms, social support and religious coping predicted post-traumatic growth.	*n* = 300 Mean age: 21.29	Botswana	Grant from the John Templeton Foundation (Grant 44040)	Drought	PTSD symptoms Post-traumatic growth	Drought exposure was associated with PTSD symptoms
Shannonhouse et al. (2019)	Correlational Purpose: To investigate the impact of religious or spiritual and meaning-focused coping on effects of disaster-related resource loss.	*n* = 300 Undergraduate university students	Botswana	Grant from the John Templeton Foundation (Grant 44040). Additional financial support was provided by the Georgia State University Centre for Stress, Trauma, & Resilience.	Drought	Positive and negative coping Lifetime trauma exposure Current trauma symptoms	Disaster-related losses and coping strategies predicted current trauma symptoms Coping or protective factors: positive coping buffered impact of disaster-related losses; negative coping appeared to partially mediate impact of disaster-related losses on trauma symptoms

Note: Please see the full reference list of the article, Mulondo M, Carter T, Thompson M, Ndlovu S. Influence of climate change on adolescents and young adults’ mental health in Southern Africa: Scoping review. Afr J Prm Health Care Fam Med. 2026;18(1), a5154. https://doi.org/10.4102/phcfm.v18i1.5154 for more information.

UNICEF, United Nations International Children’s Emergency Fund; UK, United Kingdom.

### Ethical considerations

The study received ethical approval from the University of the Free State under ethics number UFS-HSD2023/0356/2803.

## Results

**FIGURE 1:**
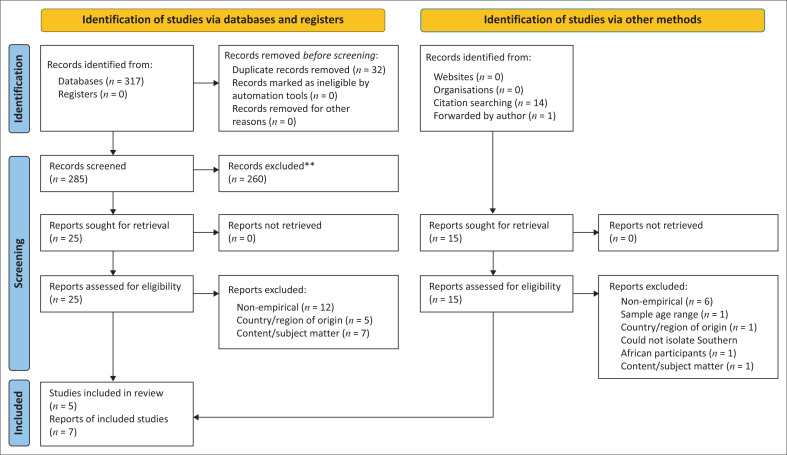
Preferred Reporting Items for Systematic reviews and Meta-Analyses diagram.

The database search resulted in 317 titles and abstracts. Grey literature search included unpublished theses and non-empirical works that were accessed through the identified databases but did not result in any additional included studies. Once duplicates were removed, 285 studies remained which were then screened for eligibility. Of the titles and abstracts screened, 26 studies were included from the original search along with 12 additional studies drawn from search the bibliography and references in empirical and reviewing articles from the study pool. After screening the full text, seven studies were included in the review. This is indicated in the PRISMA flowchart (Figure 1).

### Study characteristics

**FIGURE 2:**
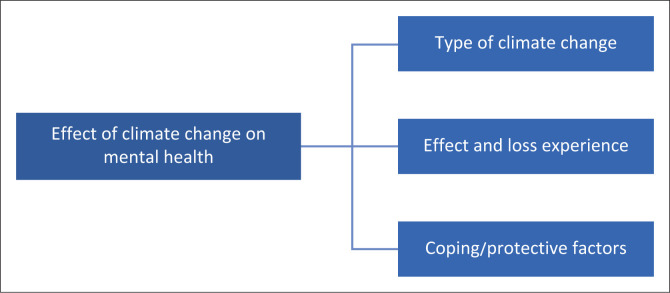
Mapping framework for effect of climate change on mental health.

All studies took place in Southern Africa, specifically Botswana (2), South Africa (1), Zimbabwe (1), Namibia (1) and Tanzania (2). The most used study design was a cross-sectional design (*n* = 4, 67%). The age group of participants in the studies is from 8 years to 23 years. The types of climate change were droughts and floods (*n* = 5, 83%) and increased temperature (*n* = 1, 17%). A majority of the mental health effect and loss experiences were characterised as post-traumatic stress disorder (PTSD) (*n* = 2, 33%), followed by traumatic symptoms (*n* = 1, 17%), depressive symptoms (*n* = 1, 17%), fatigue or low concentration and sleepiness (*n* = 1, 17%) and not specified (*n* = 1, 17%). The studies did not indicate clear health promotion interventions, instead coping strategies could be classified as personal positive coping strategies (*n* = 2; 33%) and social support and religious support (*n* = 2; 33%), while two studies did not specify any health promotion interventions (Table 1). Figure 2 provides an overview per study of the associations of climate change with mental health.

## Discussion

In this article, we gave an overview of the effects of climate change on adolescents and young adults’ mental health in Southern Africa. We found that the type of climate change event has the potential to elicit a particular mental health response from adolescents and young adults, for example, drought and floods could lead to depressive symptoms and high temperatures could lead to cognitive malaise. The mental health response also appears to be extended with cases of PTSD symptoms. Climate change is associated with various mental health experiences, such as emotional responses (sadness), heightened suicide ideation, ecological grief and loss of one’s identity.^26,27,28^ There are potential long-term impacts of climate change on children’s mental health, such as stress which is more likely to be detrimental to children’s mental health later.^3,6^

### Climate change factors contributing to mental health

Findings showed that various climate factors (EWEs) contribute significantly to increased mental health challenges among adolescents and young people, and these include drought, floods and increased temperature in adolescents and young adults. Changes in temperature has been found to be another contributing factor to increased mortality rate in populations, and poses a much higher risk on child death rate.^29^ A South African study by Subramaney et al.^30^ on climate change and mental health showed a positive relationship between high temperatures and aggression and violent behaviour. A systematic review study by Stanke et al.^31^ on various countries, including some Southern African countries, revealed that drought exposure led to increased under-5 mortality rate, under nutrition and underweight and severe wasting, particularly among children aged between 1 year and 5 years. These findings are further supported by Vins et al.^32^ who revealed the severe health consequences of exposure to drought. Furthermore, these EWEs cause extreme hard in the lives of adolescents and young adults because of their family home, school physical and economic unpleasant circumstances.^33^ Additionally, there is a resultant force of inadequate food and water supply, increased conflict risk and ecological loss.^33^ These impacts are more often than not prevalent in low-income and middle-income countries (LMICs), and this includes countries in the Southern Africa^34^ as well as those with low socioeconomic status (SES).^30^ With all the above said, experiences of such EWEs by adolescents and young adults often result to climate anxiety, which is seen through various psychological effects on their mental health, as found by Crandon et al.^35^ It is important to note that there remains a paucity of literature of EWEs impact on the lives of adolescents and young adults in the Southern African region, and this remains an important area of exploration.

### Consequences in South Africa

Climate change greatly influences the frequency and seriousness of occurrence of the previously mentioned hazardous EWEs.^36^ Children, youth and females have been found to be the most populations susceptible to the severe climate-related hazards impacts,^36^ and this is the case in South Africa, including other populations, such as those in rural areas and those who reside near power plants as reported by Barnwell.^37^ Literature further asserts that adolescents and the youth suffer the direct (injuries, poverty and death) and indirect (disruption to access to children services and conflict and migration) impacts as a result of EWEs that disrupt their daily living.^33,38^

### Psychological impacts of climate change on adolescents and young adults’ mental health

Findings of this study revealed that adolescents and young adults experience various severe psychological effects as a result of climate change. These findings are supported by a South African study by Subramaney et al.,^30^ and found that climate changes poses serious psychological threats to people who have been exposed to life-threatening circumstances such as floods, high temperatures and droughts, are susceptible to acute stress and PTSD; anxiety or psychosis or sleeping disorders; obsessive compulsive disorder (OCD); affective or mood disorders; substance use disorders; and suicide. Depression and domestic violence were also noted as psychological impacts increased by storms.^5,6^ It is possible that these influences of EWEs capture both direct physiological processes as well as the psychological impact of traumatic events and losses. There is a need to conduct research to be able to understand the experiences of climate change and mental health of adolescents and young people from their perspective in Southern Africa to make sense of the psychological impacts of climate change on their mental health.

### Health promotion interventions

According to the study findings, there were no clear or specific health promotion interventions that are currently implemented by Southern Africa countries in respect to alleviating the impact of climate change on mental health of adolescents and young adults. Nkrumah^39^ advocates for active inclusion and empowerment of young adults in climate adaptation, resilience and mitigation engagements to help derive adolescent and youth-specific appropriate and effective strategies to curb the burden of mental health as a result of climate change. Global literature speak to the importance of focusing on the long-term effects of climate change of adolescents^38^; engagement of young climate activists in policy-making and how this helps address mental health challenges as a result of climate change.^40^ Furthermore, there is a need for developing effective and appropriate coping strategies and social support for adolescents and young adults as they relate to climate change.^41^ There is a further need to consider the experiences and perceptions of adolescents and young adults to best derive context-and-age specific climate-related intervention strategies in various communities in Southern Africa. Additionally, there remains a need for more research on the protective factors of adolescents and young adults regarding climate change and its impact on mental health in Southern Africa.

This review was not without any limitations. Methodologically, some literature sources indicated ‘children’ instead of ‘adolescents’ and the ages differ per countries. There remains a need for reviews that focus on each identified EWE and its relation to climate change and mental health among adolescents and young people and not always review all EWEs under one review as this limits the depth of reporting on each EWEs. There was a further lack of literature sources from most countries in Southern Africa, and this limited the depth of this review. Strengths of this review included a rigorous review process through applying the PRISMA-ScR guidelines, which enabled authors to identify appropriate and relevant articles to include and exclude in the final synthesis.

## Conclusion

Climate change remains one of the most prevalent global public health challenges and poses threats to humanity. Climate change is also a psychological and not just an environmental challenge. There is a growing attention with regard to the impact of climate change on mental health. Adolescents and young adults are also susceptible to the consequences of climate change. The EWEs are said to be the entry point in which children’s mental health and overall health are affected by climate change, particularly those who reside in at-risk areas. There remains a literature gap on health promotion interventions as it pertains to how young people cope with climate change as one of the main psychological stressors. Furthermore, there is a need for further research to be conducted in South Africa and other LMICs.
